# The HIV Matrix Protein p17 Subverts Nuclear Receptors Expression and Induces a STAT1-Dependent Proinflammatory Phenotype in Monocytes

**DOI:** 10.1371/journal.pone.0035924

**Published:** 2012-04-30

**Authors:** Barbara Renga, Daniela Francisci, Claudio D'Amore, Elisabetta Schiaroli, Andrea Mencarelli, Sabrina Cipriani, Franco Baldelli, Stefano Fiorucci

**Affiliations:** 1 Dipartimento di Medicina Clinica e Sperimentale, University of Perugia, Perugia, Italy; 2 Dipartimento di Medicina Clinica e Scienze Biochimiche, University of Perugia, Perugia, Italy; University of Nebraska Medical Center, United States of America

## Abstract

**Background:**

Long-term remission of HIV-1 disease can be readily achieved by combinations of highly effective antiretroviral therapy (HAART). However, a residual persistent immune activation caused by circulating non infectious particles or viral proteins is observed under HAART and might contribute to an higher risk of non-AIDS pathologies and death in HIV infected persons. A sustained immune activation supports lipid dysmetabolism and increased risk for development of accelerated atehrosclerosis and ischemic complication in virologically suppressed HIV-infected persons receiving HAART.

**Aim:**

While several HIV proteins have been identified and characterized for their ability to maintain immune activation, the role of HIV-p17, a matrix protein involved in the viral replication, is still undefined.

**Results:**

Here, we report that exposure of macrophages to recombinant human p17 induces the expression of proinflammatory and proatherogenic genes (MCP-1, ICAM-1, CD40, CD86 and CD36) while downregulating the expression of nuclear receptors (FXR and PPARγ) that counter-regulate the proinflammatory response and modulate lipid metabolism in these cells. Exposure of macrophage cell lines to p17 activates a signaling pathway mediated by Rack-1/Jak-1/STAT-1 and causes a promoter-dependent regulation of STAT-1 target genes. These effects are abrogated by sera obtained from HIV-infected persons vaccinated with a p17 peptide. Ligands for FXR and PPARγ counteract the effects of p17.

**Conclusions:**

The results of this study show that HIV p17 highjacks a Rack-1/Jak-1/STAT-1 pathway in macrophages, and that the activation of this pathway leads to a simultaneous dysregulation of immune and metabolic functions. The binding of STAT-1 to specific responsive elements in the promoter of PPARγ and FXR and MCP-1 shifts macrophages toward a pro-atherogenetic phenotype characterized by high levels of expression of the scavenger receptor CD36. The present work identifies p17 as a novel target in HIV therapy and grounds the development of anti-p17 small molecules or vaccines.

## Introduction

Matrix proteins are essential in the viral replication cycle. The major structural protein of all retroviruses is a multidomain polypeptide called Gag that is capable of assembling into virus-like particles in the absence of other viral constituents [1]–[4]. The HIV-1 Gag is synthesized as a precursor polyprotein, Pr55Gag, which consists of four major domains cleaved by the virally-encoded protease into its mature products p17 matrix, p24 capsid, p7 nucleocapsid, the carbossi (C)-terminal p6, and several small polypeptides including p1 and p2 [1]. The HIV-1 matrix protein p17 encompasses the amino (N)-terminal domain of the gag gene and, in mature virions, is a 132-AA polypeptide myristoylated at the N-terminus which forms a protective shell associated directly with the inner leaflet of the viral membrane [1]–[4]. The P17 serves several function in the viral replication cycle including viral nuclear import at early stage of infection. In the late stage, p17 mediates the recruitment of the viral surface/transmembrane gp120/gp41 envelope protein complex into virions and targets Pr55Gag proteins to their assembly sites at the plasma membrane of infected cells [1]–[4]. In addition to its role in viral replication, HIV-1-infected cells release significant amounts of virion-free p17. This exogenous p17 is detected in the plasma of HIV-1-infected persons at nanomolar concentrations [5] and the protein might accumulate in the germinal center of lymphonodes in HIV infected patients receiving an highly effective antiviral terapy (HAART) [6]–[9]. P17 dysregulates biological activities of different immune cell types that are directly or indirectly involved in AIDS pathogenesis (i.e. T lymphocytes, monocytes and dendritic cells) [10]. These activities occur after p17 interaction with a cell surface receptor (p17R) which is expressed by a definite subset of immune cells [10]. The nature of this receptor is still unknown, althought p17 binds to heparan sulphate side chains of syndecan-2, syndecan-4 and CD44v3 and these heparan sulphate proteoglycans colocalize with HIV-1 p17 on activated human CD4+ T cells [11].

Monocytes/macrophages play an important role in HIV infection. Thus, while less susceptible than CD4+ T lymphocytes to viral cytopathic effects, they are resistant to HIV-1-induced apoptosis [12] and therefore serve as a major virus reservoir [13]. The analysis of HIV-infected monocytes shows activation of multiple signalling cascades leading to up-regulation of variety of inflammation-related molecules that might sustain both inflammation and HIV-1 replication [14], [15]. Molecular dissection of the relative contribution of p17 to this phenotype has revealed that exposure of human monocytes, that constitutively express the p17R, to p17 triggers the release of MCP-1 [16] a chemokine whose regulation is achieved through the activation of the signal transducers and activators of transcription (STAT)-1 pathway [17], which is prominent in regulating chemokines mediated inflammatory responses and has been implicated in the pathogenesis of HIV infection and disease progression [18]–[20].

In addition to their role in immune response, monocytes exert metabolic functions [21], [22]. Reciprocal regulation of immune metabolic activities in these cells is achieved by the activities of member of the nuclear receptors superfamily [23]–[25]. Thus, modulation of peroxisome proliferator-activated receptor (PPAR) α and γ, liver–x-receptor (LXR) α and β, farnesoid –x-receptor (FXR), vitamin D receptor (VDR) in these cells triggers a reciprocal regulation of immune and metabolic pathways [24]–[27]. Because the activity of these nuclear receptors is anti-inflammatory in nature, their expression is often downregulated in monocytes undergoing immune activation [28]. In the present study we provide evidence that the HIV-1 p17 matrix protein shifts macrophages to a pro-inflammatory and pro-atherogenic phenotype and that this effects is reached through the downregulation of the expression/function of PPARγ and FXR. Because these effects are achieved by the highjacking of a Rack-1/Jak-1/STAT-1 pathway and are reversed by STAT-1 inhibition, this study identify novel targets for the treatment of immune activation and metabolic dysfunction in virologically suppressed HIV-infected persons.

## Results

### p17 treatment regulates macrophage functions in humans

**Figure 1:**
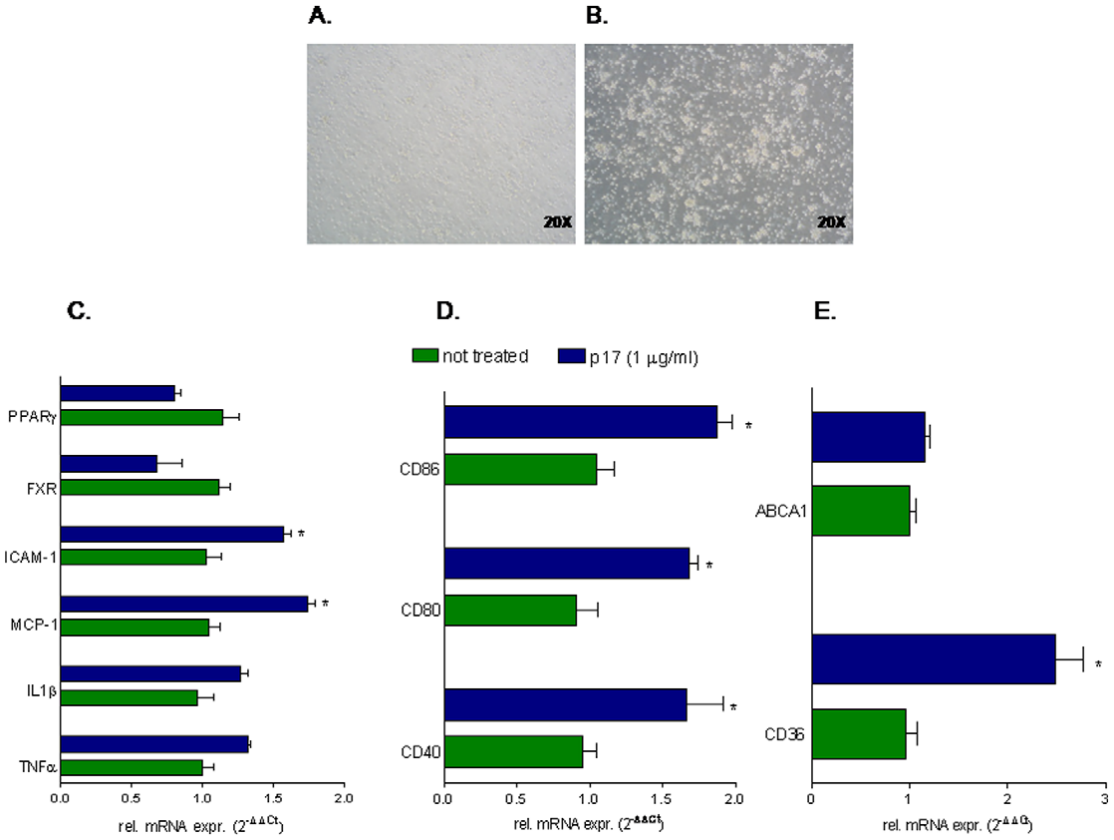
HIV-1 p17 regulates pro-inflammatory, co-stimulatory and pro-atherogenic molecules in CD14-derived PBMC isolated from healthy donors. CD-14 derived PBMC isolated from healthy donors were stimulated for 18 hours with 1 µg/ml p17 recombinant protein. (A and B) Exposure to p17 drives an activated phenotype in macrophages and causes macrophages adhesion. Magnification 20×. (C) Relative mRNA expression of proinflammatory cytokines TNFα, IL1β, MCP-1, ICAM-1 and nuclear receptors FXR and PPARγ. (D) Relative mRNA expression of co-stimulatory molecules CD40, CD80 and CD86. (E) Relative mRNA expression of proatherogenic genes CD36 and ABCA1. Real-Time analysis was carried out in triplicate and the experiment was repeated twice. *P<0.05 versus not treated cells.

To characterize the effect of p17 on macrophage activation, CD14-derived peripheral blood mononuclear cells (PBMC) from healthy donors were stimulated 18 hours with 1 µg/ml p17. As shown in Figure 1A and B, p17 treatment caused a significant monocytes aggregation as documented by phase-contrast microscopy analysis of these cells. This phenotype was in accordance with Real-Time PCR data showing an increased expression of the adhesion molecule ICAM-1 following p17 stimulation (Fig. 1C). In addition, exposure to p17 caused a robust increase in the expression of proinflammatory mediators including MCP-1 as well as of co-stimulatory molecules such as CD80, CD86 and CD40 (Fig. 1C and D). This inflammatory phenotype associated with a dysregulation of the expression of genes involved in lipid metabolism. Thus, p17 increased CD36 mRNA expression while failed to boots ABCA1 (Figure 1E). Since macrophages activation results in a reciprocal regulation of immune response and lipid metabolism and this integration is reached to the activation of members of the nuclear receptor superfamily (e.g. FXR and PPARγ) [23]–[27], we have investigated whether p17 stimulation regulates the expression of these transcription factors. As shown in Figure 1C, a slightly down-regulation of FXR and PPARγ was found in CD14-derived PBMC following stimulation with p17, unrevealing a novel role for this viral protein in regulating lipid metabolism and immune responses these cells.

### Biological activities of p17 were almost completely neutralized by therapeutical vaccination

**Figure 2:**
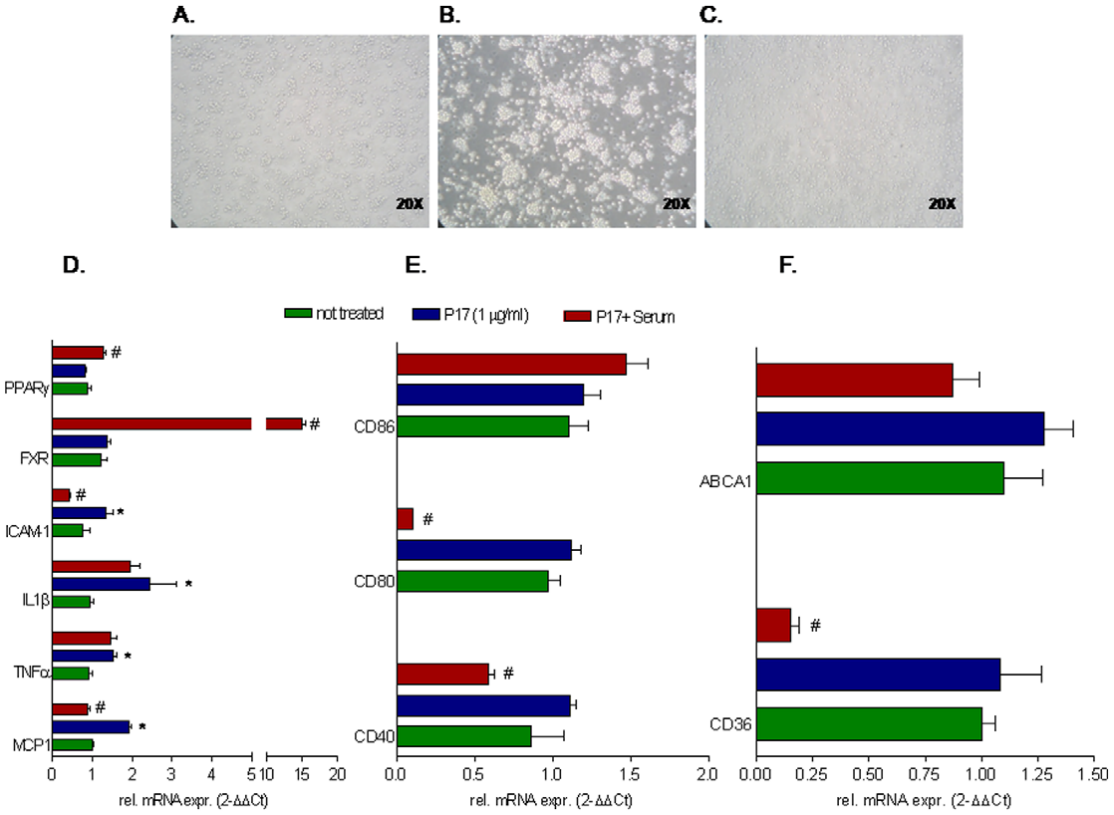
HIV-1 p17 exerts regulatory effects on CD-14 derived PBMC isolated from HIV infected patients. CD-14 derived PBMC isolated from HIV infected patients vaccinated with an anti-p17 vaccine were stimulated ex vivo with 1 µg/ml p17 for 18 hours with or without the serum of the same patients (diluted 1∶100 in medium culture). (A–C) Activation of CD-14 derived PBMC caused by p17 (b) was reserved by p17 immune-neutralization with anti-p17 serum. Magnification 20×. (D) Relative mRNA expression of proinflammatory cytokines TNFα, IL1β, MCP-1, ICAM-1 and nuclear receptors FXR and PPARγ. (E) Relative mRNA expression of co-stimulatory molecules CD40, CD80 and CD86. (F) Relative mRNA expression of proatherogenic genes CD36 and ABCA1. Real-Time analysis was carried out in triplicate and the experiment was repeated twice. *P<0.05 versus not treated cells. #P<0.05 versus p17 stimulated cells.

Having shown that p17 protein regulates the expression of nuclear receptors as well as that of genes involved in cell adhesion, inflammatory response and lipid metabolism we have investigated whether these effects could be reproduced in CD14-derived PBMC isolated from virologically suppressed HIV infected persons receiving HAART. For these studies CD14-derived PBMC cells were stimulated ex vivo with 1 µg/ml p17. As shown in Figure 2 A, B and C, p17 treatment induces monocytes aggregation and effectively increased the expression of pro-inflammatory genes, including MCP-1, TNFα, IL-1β and ICAM-1 (Fig. 2B). To investigate whether these effects of p17 were receptor specific, we took advantage from the availability of sera obtained from HIV-infected patients vaccinated with p17 peptide. These sera contain neutralizing p17 antibodies (Method S1 and Figure S1). Of interest p17 neutralization with p17 antisera effectively reversed the stimulatory effects of p17 on macrophages (Fig. 2C). Indeed, exposure to anti-p17 retained in sera of the vaccinated patients almost completely abrogate the effects of p17 in terms of induction of MCP-1, ICAM-1, CD36, CD80 and CD40 but were not able to counter-regulates the induction of CD86, TNFα and IL1β (Fig. 2 D, E and F). In contrast to the effects seen in healthy donors, p17 treatment failed to down-regulate the expression of FXR and PPARγ in CD14-derived PBMC isolated from HIV patients while p17 immune-neutralization significantly induced the expression of these nuclear receptors, further supporting the view that p17 is a negative regulator of FXR and PPARγ. All together these data confirmed that some of the effects of p17 observed in CD14-derived PBMCs obtained from healthy donors were maintained in HIV infected macrophages and that anti-p17 immune-neutralization leads to a specific reversion on these activities.

### p17 treatment regulates macrophage functions in THP-1 cells

**Figure 3:**
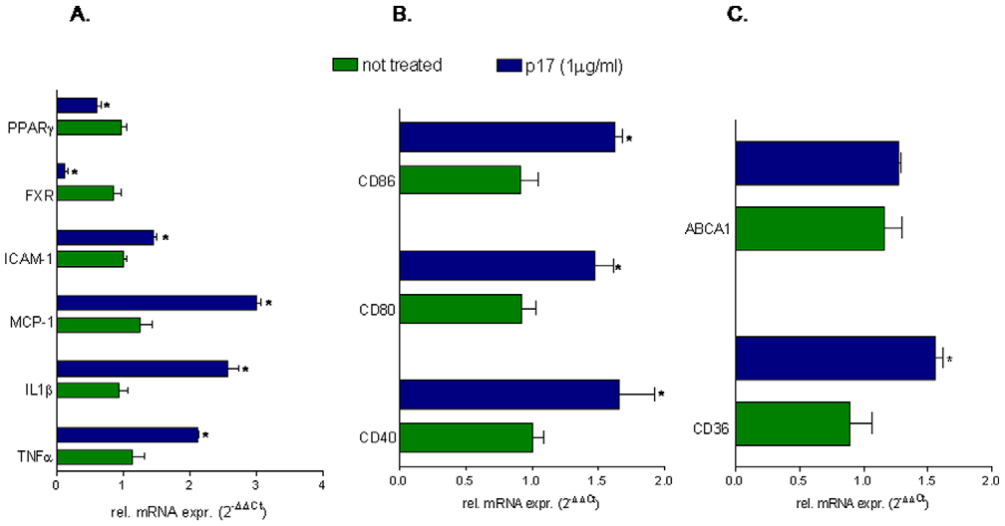
Stimulation of THP-1 cells with p17 causes reciprocal regulation of genes involved in immune function and lipid metabolism. THP-1 cells were stimulated with 1 µg/ml p17 recombinant protein for 18 hours. (A) Relative mRNA expression of proinflammatory mediators TNFα, IL1β, MCP-1, ICAM-1 and nuclear receptors FXR and PPARγ. (B) Relative mRNA expression of co-stimulatory molecules CD40, CD80 and CD86. (C) Relative mRNA expression of proatherogenic genes CD36 and ABCA1. Real-Time analysis was carried out in triplicate and the experiment was repeated twice. *P<0.05 versus not treated cells.

The signalling pathways mediating the effects of p17 on macrophages were investigated by using THP-1 cells, a myeloid monocytic cell line. As expected, exposure of these cells to p17 (1 µg/ml) caused a robust increase in the expression of ICAM-1, MCP-1, TNFα and IL1β mRNA as well as of co-stimulatory molecules CD40, CD80 and CD86 (Fig. 3A and B). The analysis of genes involved in lipid metabolism revealed that p17 stimulation effectively increases CD36 but not ABCA1 mRNA. In addition, exposure of THP1 cells to p17 caused a robust downregulation of FXR and PPARγ mRNA (Fig. 3A and C).

### p17 induces STAT1 phosphorylation in THP-1 cell line

**Figure 4:**
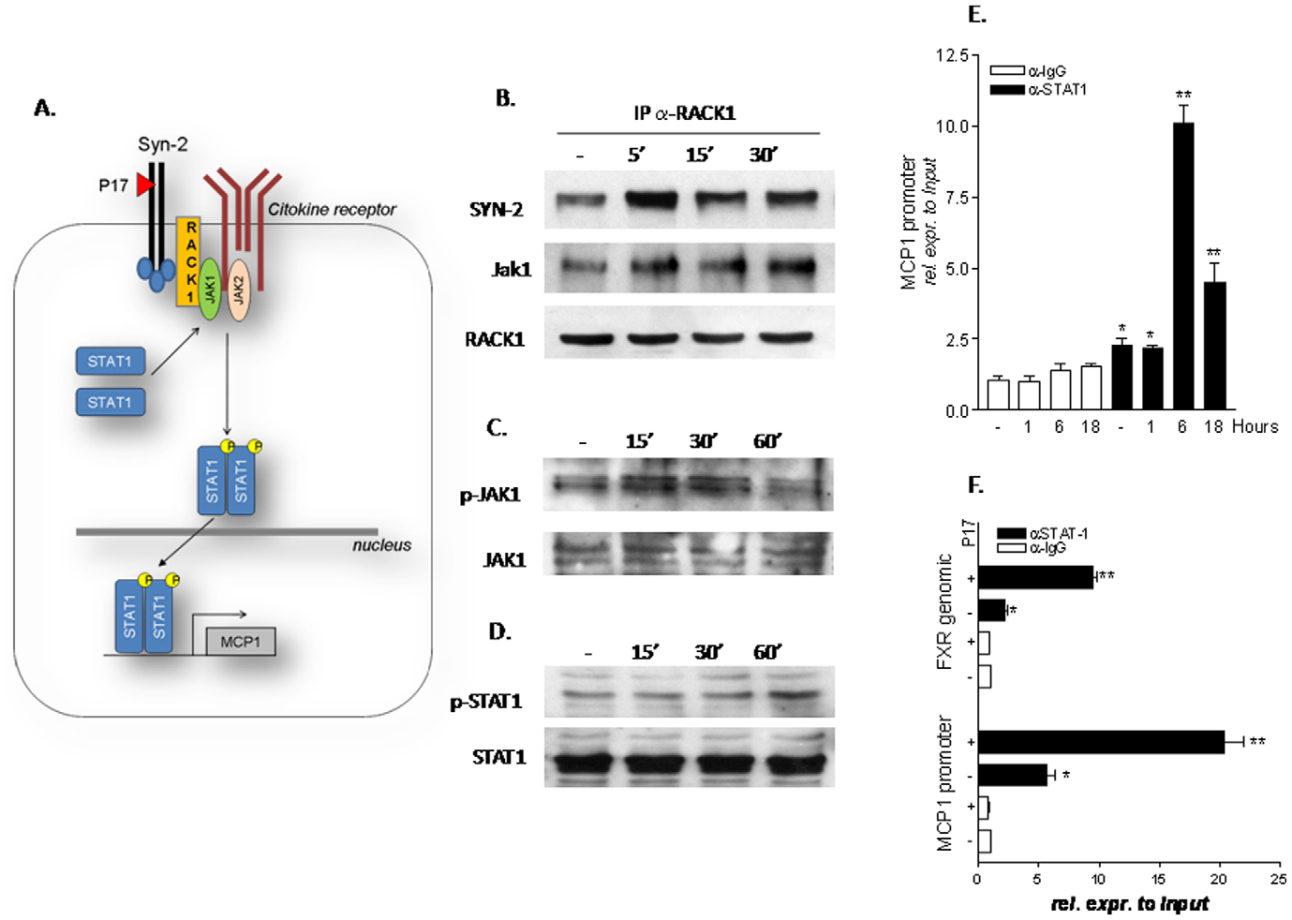
p17 signals through RACK-1/Jak-1/STAT-1 pathway in THP-1 cells. (A) Schematic diagram showing putative interaction of p17 with syndecan-2 and the activation of RACK-1/Jak-1/STAT-1 signal transduction pathway. (B) Co-immuneprecipitation of RACK-1 with syndecan-2 and Jak-1 following stimulation of THP-1 cells with p17 recombinant protein (1 µg/ml) for 5, 15 and 30 minutes. (C) Analysis of Jak-1 protein (total and phosphorylated fraction) by immune blot in THP-1 cells stimulated with p17 (1 µg/ml) for 15, 30 and 60 minutes. (D) Analysis of STAT-1 protein (total and phosphorylated fraction) by immune blot in THP-1 cells stimulated with p17 (1 µg/ml) for 15, 30 and 60 minutes. (E) Chromatin Immunoprecipitation assay carried out in THP-1 cells left untreated or primed with 1 µg/ml p17 for 1, 6 and 18 hours. Real-Time PCR was performed on MCP-1 promoter. (F) Chromatin Immunoprecipitation assay carried out in THP-1 cells left untreated or primed with 1 µg/ml p17 for 18 hours. Real-Time PCR was performed on both MCP-1 promoter and intron-II of FXR gene. Values are normalized relative to input DNA concentration and are expressed relative to those of not treated cells immunoprecipitated with an anti IgG antibody, condition set as 1. Analysis was carried out in triplicate and the experiment was repeated twice. *P<0.05 versus not treated cells. **P<0.05 versus p17 stimulated cells.

Previous studies have shown that the expression of MCP-1, ICAM-1, CD40, CD86 and CD36 is induced in STAT-1 dependent manner while, in inflamed cells, STAT-1 activation results in down regulation of FXR and PPARγ gene expressions [29]–[31]. Because the STAT-1 pathway could be activated through a Rack-1/Jak-1 pathway and p17 binds to syndecan-2, a glycoprotein that effectively recruits Rack-1 to a multiprotein complex at the cell membrane [11], [32]–[33] we then investigated whether exposure of THP1 cells to p17 activates this pathway (Figure 4A). For this purpose lysates from THP-1 cells left untreated or primed with p17 were immunoprecipitated with an anti-Rack-1 antibody and immunoblotted with antibodies against Syndecan-2 and Jak-1. Results from this experiment demonstrated that p17 stimulation recruits Rack-1 into a syndecan-2/Rack-1 multiprotein complex within 5 min of exposure and that this protein-protein complex dissociates after 15 and 30 minutes (Figure 4B). This multiprotein complex is also characterized by the recruitment of Jak-1, though the interaction of the later protein with syndecan 2 and Rack-1 is maintained up to 30 minutes (Figure 4B). The results of these experiments were consistent with results from Western blotting analysis of the time-course of Jak-1 phosphorylation. Indeed, Jak-1 phosphorylation, a measure of Jak-1 activation, occurs between 15 and 30 minutes exposition to p17, but decreases thereafter (Figure 4C). Furthermore, exposure of THP-1 cells to p17 resulted in STAT-1 phosphorylation that became apparent after 60 min of incubation with 1 µg/ml p17. All together, these data support the notion that p17 signals through a pathway involving syndecan-2/RACK-1/Jak-1 and STAT-1.

Since previous studies have demonstrated that the promoter of MCP-1 as well as the intron-II of FXR gene are regulated in a STAT1 dependent manner [19], [30] we have then examined whether p-17 mediated activation of STAT-1 regulates the transcription of these genes. Thus, as illustrated in Figure 4E, kinetic of MCP-1 promoter occupancy revealed that the binding of STAT-1 peaked at 6 hours and was maintained until 18 hours treatment of THP-1 cells to p17. We have repeated the ChIP and demonstrated that, similar to the MCP-1 promoter, STAT-1 also bound to the intron-II of FXR gene after 18 hour-stimulation with p17.

### STAT-1 inhibitor fludarabine reverses p17 effects on macrophages

**Figure 5:**
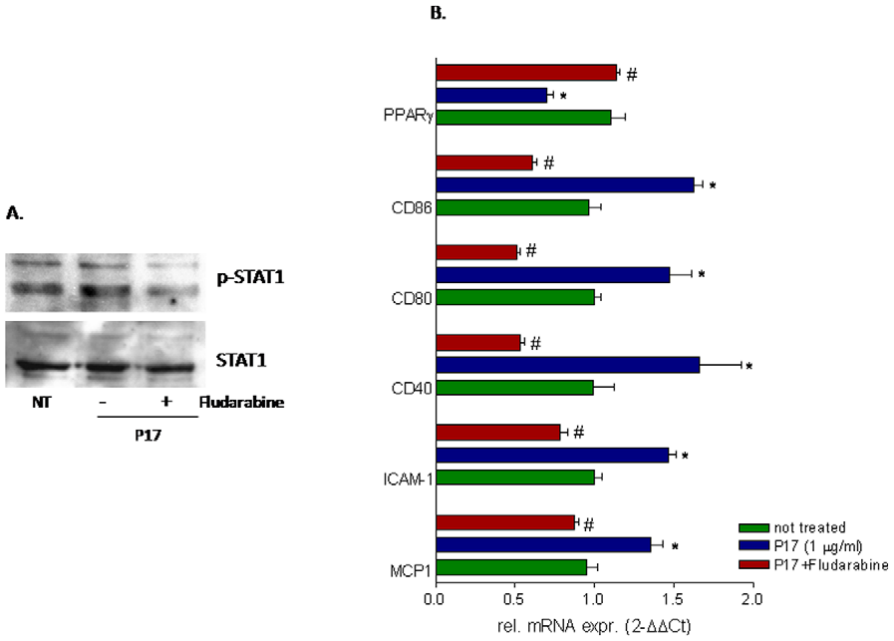
An intact STAT-1 signal is required to mediate the biological activity of p17. THP-1 cells were stimulated for 18 hours with p17 (1 µg/ml) in presence or in absence of a specific STAT-1 inhibitor fludarabine (0.5 µM). At the end of treatments cellular lysates were used for Real-Time or immunoblot analysis. (A) Immunoblot of STAT-1 protein (total and phosphorylated fraction). (B) Relative mRNA expression of MCP-1, ICAM-1, PPARγ, CD40, CD80 and CD86 was expressed relative to not treated cells. Analysis was carried out in triplicate and the experiment was repeated twice. *P<0.05 versus not treated cells. #P<0.05 versus p17 stimulated cells.

To demonstrate the relevance of STAT-1 in the context of the regulatory activity exerted by p17 in macrophage's cell lines, we pretreated THP-1 cells with fludarabine, a STAT1 inhibitor [34], before the challenge with p17. As illustrated in Figure 5A, p17 mediated STAT-1 phosphorylation was inhibited by fludarabine co-treatment, and exposure to this agent completely reversed regulatory effects exerted by p17 on MCP-1, ICAM-1, CD40, CD80, CD86 and PPARγ.

### Activation of nuclear receptors counter-regulates the biological activities of p17

**Figure 6:**
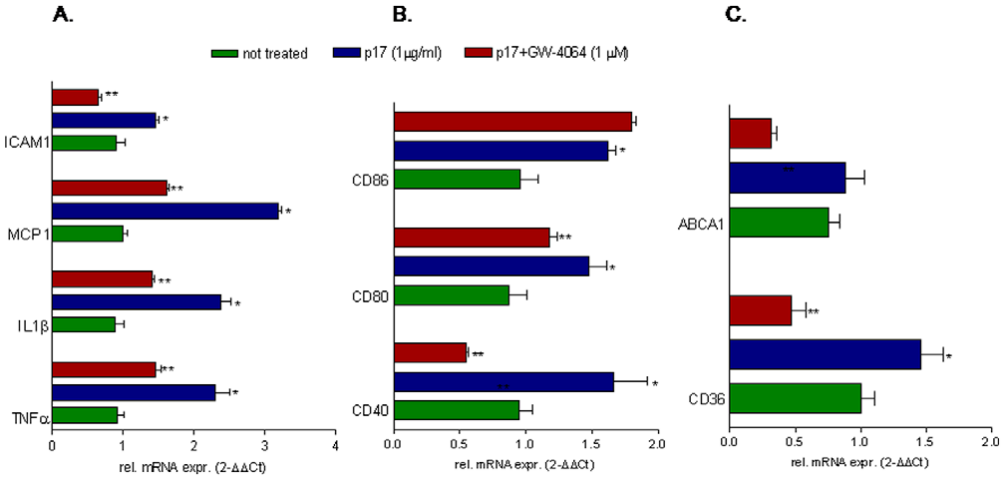
FXR agonist, GW-4064, reverses the effects exerted by p17 on THP-1 cells. THP-1 cells were pre-incubated 2 hours with GW-4064 (1 µM) before administration of p17 (1 µg/ml for 18 hours). Total RNA was extracted and the relative mRNA expression of (A) proinflammatory cytokines and nuclear receptors, (B) co-stimulatory molecules and (C) proatherogenic genes was assayed by Real-Time PCR. Values are expressed relative to not treated cells. Analysis was carried out in triplicate and the experiment was repeated twice. *P<0.05 versus not treated cells. **P<0.05 versus p17 stimulated cells.

**Figure 7:**
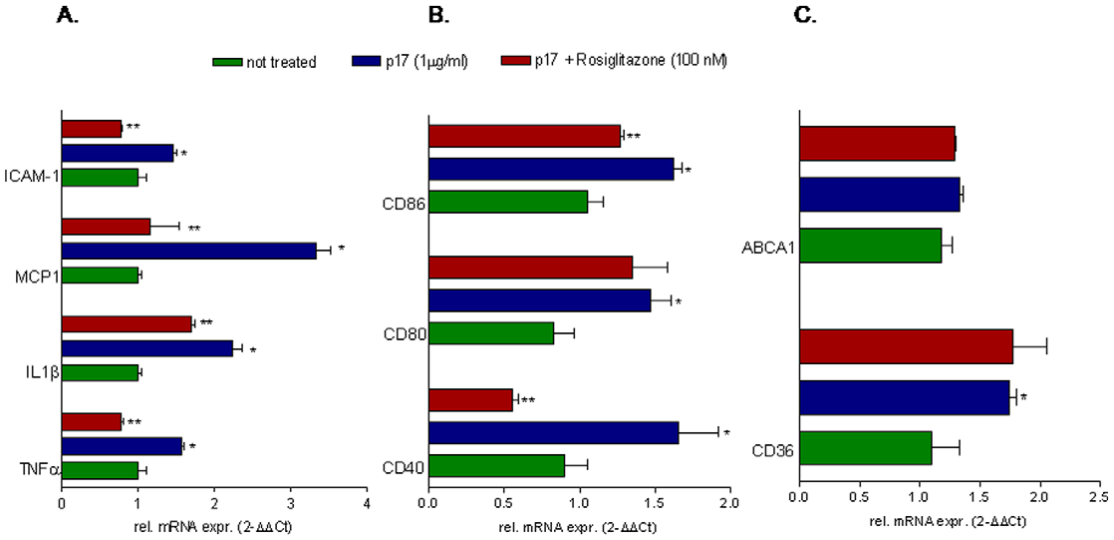
The PPARγ agonist, rosiglitazone, differentially regulates p17 biological activities. THP-1 cells were pre-incubated 2 hours with rosiglitazone (1 µM) before administration of p17 recombinant protein (1 µg/ml for 18 hours). Total RNA was extracted and the relative mRNA expression of (A) proinflammatory cytokines and nuclear receptors, (B) co-stimulatory molecules and (C) proatherogenic genes was analyzed by Real-Time PCR. Values are expressed relative to not treated cells. Analysis was carried out in triplicate and the experiment was repeated twice. *P<0.05 versus not treated cells. **P<0.05 versus p17 stimulated cells.

Since FXR and PPARγ exert their regulatory activities at the interface between inflammation and metabolism and their expression is negatively regulated by exposure to p17, we have then speculated whether ligands for these transcription factors could reverse the effects of p17. For this purpose THP-1 cells were incubated with p17 alone or in the presence of GW-4064 or rosiglitazone, two selective agonists for FXR and PPARγ. As shown in Figure 6, FXR activation with GW4064 effectively down-regulated TNFα, IL1β, ICAM-1, MCP-1,CD40 and CD80 induction caused by p17 (Figure 6A), but failed to reverse induction of CD86 (Fig. 6B). Furthermore the FXR agonist significantly reverted regulation of CD36 and ABCA1 mRNA caused p17 (Figure 6C). Similarly to the FXR agonist, the PPARγ ligand rosiglitazone counteracted induction of TNFα, IL1β, ICAM-1, MCP-1, CD40 and CD86 (Figure 7A) caused by p17 but failed to do the same on CD80 (Fig. 7B). Additionally, PPARγ activation failed to reverse the induction of CD36 mRNA caused by p17. This was not surprising because PPARγ is a well know inducer of CD36 gene expression [35].

## Discussion

Persistent immune activation and lipid dysmetabolism occur often in virologically suppressed HIV infected persons taking HAART and are thought to make a contribution to long-term effects linked to chronic HIV infection [36]. Because HAART *per se* might induce hypetrglyceridemia, the current view is that concomitant effects derived from HIV infection *per se* and side effects due to HAART are both causative factors for the increased risk of accelerated atherosclerosis and ischemic cardiovascular events in long term survival HIV-infected persons [37]. Beside the role of HAART, however, the virus infection *per se* has been shown to drive a chronic state of subclinical inflammation in successfully treated patients. Since under HAART more than 99% of HIV-1 particles detected in the circulation are not productively infectious virions, these particles as well as circulating viral proteins should make a contribute to these complications [38]–[42]. The p17 HIV-matrix protein is a structural protein whose biological functions are deemed essential for virus life cycle [4]. In addition, p17 displays a variety of biological activities outside the infected cells [4]. Despite molecular mechanisms mediating these effects are incompletely identified, the role of p17 in maintaining chronic inflammation in virologically suppressed patients should not be minor, because the presence of anti-p17 neutralizing antibodies in the plasma of HIV infected patients correlates with a better prognosis and slows the disease progression [43].

Confirming a common theme in chronic infection (i.e. the ability of viral proteins to pirate molecular targets in mammalian cells) the HIV p17 has been shown to interact with putative binding sites on activated CD4+ and CD8+ T cells as well as on NK cells [10]–[11]. Despite the nature of this p17R is still under investigation, exposure to p17 triggers a complex system of second messengers and leads to an activated phenotype of immune cells [44].

In the present study we provided evidence that p17 exerts its regulatory function on macrophages by simultaneously interaction with immune and metabolic activities of these cells. Because macrophages exert an essential role in both innate immunity and lipid metabolism and lipid-engulfed macrophages (foam cells) are prototypical to atherosclerotic plaques [45], our data establish a robust link between the modulation of these cells and lipid dysmetabolism observed in HIV infected persons. Despite the cell surface receptor of p17 in macrophages was not defined in the present study, we have provided robust evidence that such a target does exist. Indeed, taking advantage of sera obtained from HIV infected persons enrolled in a phase I trial testing the safety of an anti-p17 vaccine, we have found that immune neutralization of p17 with sera of these patients completely reverts the regulatory functions of the recombinant protein in macrophages.

While numerous studies have investigated the role of anti-HIV drugs in lipodystrophy and dyslipidemia [37], the effects of HIV infection on cellular cholesterol metabolism remain poorly characterized. Recently, a role for the CD36 scavenger receptor as well as for the ATP-binding cassette transporter A1 (ABCA1) in the genesis of HIV-related dyslipidemia has been propsed [46], [47]. In macrophages CD36 mediates the uptake of oxidized low-density lipoproteins (ox-LDLs). Thus, the expression of CD36 is essential to cholesterol accumulation in these cells, their transition to foam cells and development of atherosclerotic lesions [48]. Conversely, the protein transporter ABCA1 is involved in cholesterol efflux from macrophages [49]. Results presented here demonstrated that the HIV-1 matrix protein p17 regulates the expression of these genes in macrophages isolated from healthy donors and HIV-infected persons treated with HAART, despite the magnitude of the reported effects was different. In these cells, p17 significantly induces CD36 mRNA expression but fails to alter the levels of ABCA1, thought that a minor increase in the expression of this gene was observed in THP1 cells. These data are consistent with previous works showing increased levels of circulating CD36 while the levels of ABCA1 are reduced or unchanged in HIV infected persons under HAART, and support the notion that HIV infection resets the expression of genes to a pattern that favours the accumulation of intracellular lipids [46], [47].

In the present study we provided evidence that exposure of macrophages to recombinant p17 effectively increases the expression of ICAM-1 mRNA. Several studies have demonstrated that circulating levels of ICAM-1 are increased in HIV-infected and acquired immune deficiency syndrome (AIDS) persons [50]. Despite regulatory cytokines such as TNFα and IL1β may contribute to the increased levels of ICAM-1 [51] we have now made the important observation that HIV matrix protein p17 contributes to the induction of this pro-inflammatory mediator. Because the role of viral proteins in resetting the immune system in HIV infection is not completely understood, we have investigated whether p17 regulates the expression/generation of prototypical cytokines in PBMC isolated from healthy subjects and HIV infected persons. Our results demonstrated that exposure of macrophages to this protein effectively increases the expression of TNFα and IL1β mRNA in macrophages isolated from HIV infected patients but not in healthy donors macrophages, suggesting that p17 signals as “co-stimulatory” molecule in this settings.

Adding to the immune-regulatory role of p17 in macrophages we provided evidence that p17 significantly increases the expression of CD40 mRNA. CD40 is a cell-surface co-stimulatory molecule involved in macrophage activation by its ligation via CD154 (CD40L) expressed on activated CD4+ T cells [52]. Ligation of CD40, drives the synthesis of pro-inflammatory cytokines including TNFα and IL1β as well as of other co-stimulatory molecules including CD80 and CD86 [52]. Consistent with the ability of p17 to reset the expression of CD40 we also observed an up-regulation of CD80 and CD86 in macrophages exposed to p17. These observations might have a translation readout since a growing body of evidences supports a role for the CD40 pathway in macrophage activation as well as in the atherosclerosis progression [53].

Another important observation we made in this study was the demonstration that p17 causes a robust decrease in the expression of two nuclear receptors, FXR and PPARγ, in THP1, while this effect was not observed in CD14-derived PMBC obtained from HIV infected persons, and macrophages from healthy subjects showed an intermediate phenotype. FXR and PPARγ are transcription factors that play a role in the regulation of lipid metabolism and immune function in monocytic cells [24]–[26]. Their regulatory activity is inhibitory in nature and therefore both receptors have been proposed as potential candidates to take control over dysregulated immune response and lipid metabolism in the setting of chronic inflammatory disorders-associated metabolic dysfunctions [27]. We have now provided evidence that an HIV viral protein has the potential to modulate the expression of these nuclear receptors and that abrogation of the expression of FXR and PPARγ is instrumental to the development of proinflammatory activity of p17 in macrophages. Support to this view comes from the observation that exposure of p17 treated macrophages to FXR and PPARγ ligands reverts the immune activated phenotype and counter-regulates the expression of metabolic genes. However, it has to be noted that the effects exerted by the FXR and PPARγ agonists differ under some aspects. Thus, while GW4064 effectively reduced p17-mediated CD40 and CD80 co-stimulatory molecules expression, it failed to reverse the induction of CD86. In contrast, rosiglitazone reduced p17-medaied CD40 and CD86 expression, but failed to reverse the induction of CD80 mRNA. Because the two ligands act on different targets these discrepancies are likely linked to their specific mode of action [24]–[27].

In the present study we have also investigated whether a common motif could support the regulatory effects p17 exerts on immune and metabolic functions in macrophages. Intracellular dissection of these signaling pathways took advantage from the demonstration that physical interaction between p17 and syndecan-2 at plasma membrane supports the activation of human CD4+ T cells exposed to the matrix protein [11]. Previous studies have shown that syndecan-2 interacts with Rack-1 [32] while phosphorylated Rack-1 is recruited in a protein complex with Jak-1 [33]. Consistent with this hypothesis, exposure to p17 causes the formation of a multiprotein complex containing syndecan-2, Rack-1 and Jak-1. In addition, we provided evidence that activated Jak-1 phosphorylates and activates STAT-1. Because STAT-1 is recruited to specific STAT-1 binding sites in the promoter regions of MCP-1, FXR and PPARγ our data provided a robust evidence that by pirating a Rack-1/Jak-1/STAT-1 pathway the HIV protein p17 takes control over immune and metabolic functions in macrophages. This view was further confirmed using fludarabine, a pharmacological inhibitor of STAT-1. Of relevance, fludarabine almost completely reversed the biological activities of p17 on macrophages.

In summary, findings presented in this study demonstrated that the HIV-1 matrix protein p17 interferes with intracellular lipid metabolism and immune function in macrophages. This study highlights the potential use of FXR and PPARγ agonists as well as of the therapeutic p17 vaccine or small molecule inhibitors as potential agents for the treatment of immune and metabolic dysfunction in HIV infection.

## Materials and Methods

### Patients

To investigate whether p17 neutralization effectively antagonizes the effects exerted by p17 on human macrophages, p17 was immune-neutralized by incubation with sera from HIV infected persons enrolled in a Phase 1 study designed to investigate the safety and immunogenicity of recombinant p17 peptide in HIV. The therapeutic vaccination was performed using a 20 amino acids peptide, named AT20 (SGGELDRWEKIRLRPGGKKK). Previous studies have shown that immunization of animals with AT20 resulted in the development of p17 neutralizing antibodies capable of blocking all biological activities of the viral protein [54]. The vaccination protocol n. MED-AT20-001 Eudract Number 2008-001465-29 was approved by the Ethical committee of Regione Umbria (Italy) on June 25, 2010 authorization n. 1558/10. Authorization for collecting and using blood samples from HIV infected persons for *ex vivo* testing was also granted by the ethical committee of Regione Umbria (Italy) on July 22, 2010 (authorization number CEAS 1654/20). An informed written consent was obtained from each participant to the study. At the end of the study blood samples were obtained from three patients and the recombinant p17 protein was immune-neutralized by pre-incubation with the serum of these patients. The presence of anti-p17 antibodies in the blood of these subjects was verified by micro-Elisa.

### Reagents

HIV-p17 recombinant protein was provided by Medestea (Torino, Italy). The FXR ligand GW-4064, the PPARγ ligand rosiglitazone and the specific STAT-1 inhibitor fludarabine were from Sigma Aldrich.

### Isolation and culture of human CD14-derived PBMC

PBMC were first isolated by density gradient centrifugation using the Hystopaque reagent (Pharmacia Biotech) and then positively selected using CD14 magnetic beads and LS columns according to the manufacturer's instructions (Miltenyi Biotec). After isolation cells were stimulated 18 hours with 1 µg/ml p17 in RPM-I supplemented with 10% Fetal Bovine serum, 1% L-glutamine and 1% penicillin/streptomycin. PBMC isolated from vaccinated HIV patients were also stimulated with the combination of p17 (1 µg/ml) and serum (diluted 1∶100 in culture medium).

### Microscopic images acquisition

Images of PBMC were acquired with a Nikon Eclipse TE300 microscope using a Nikon Digital Camera DXM1200.

### Cell culture

THP-1 cells, a human acute monocytic leukemia cell line (ATCC collection imported by Promochem, Milan, Italy), were cultured in RPM-I medium supplemented with 10% Fetal Bovine serum, 1% L-glutamine and 1% penicillin/streptomycin.

### Real-Time PCR

Total RNA was isolated from livers of cells using the TRIzol reagent (Invitrogen). RNA (1 µg) was reverse-transcribed using random hexamer primers and Super Script II reverse transcriptase (Invitrogen). mRNA were quantified by Real-Time quantitavive PCR on iCycler apparatus (Biorad) using specific primers: hGAPDH: gaaggtgaaggtcggagt and catgggtggaatcatattggaa; hFXR: tacatgcgaagaaagtgtcaaga and actgtcttcattcacggtctgat; hPPARγ: gctggcctccttgatgaata and ttgggctccataaagtcacc; hMCP1: ccccagtcacctgctgttat and tcctgaacccacttctgctt; hICAM1: agcttctcctgctctgcaac and cattggagtctgctgggaat; hIL1β: ggacaagctgaggaagatgc and tcgttatcccatgtgtcgaa; hTNFα: aacctcctctctgccatcaa and ggaagacccctcccagatag; CD40: ctcagaaacagacaccatctgc and tcagaaacccctgtagcaatct; CD80: agggaacatcaccatccaag and tgccagtagatgcgagtttg; CD86: agacgcggcttttatcttca and ccctctccattgtgttggtt; ABCA1: gcttgggaagatttatgacagg and aggggatgattgaaagcagtaa; CD36: tttctgtatgcaagtcctgat and attaagccaaagaataggcac. PCR amplifications and data analysis were performed as described [55].

### Western Blotting

1×10^6^ THP-1 cells serum starved over-night were stimulated with p17 (1 µg/ml) for 15, 30 and 60 minutes. To investigate the effects of Fludarabine (a specific STAT-1 inhibitor) THP-1 cells, serum starved over night, were stimulated with p17 (1 µg/ml) alone or in combination with Fludarabine (0.5 µM) for 18 hours. Total lysates were prepared by solubilization of cells in E1A lysis buffer containing phosphatase and protease inhibitors. Proteins were separated by polyacrylamide gel electrophoresis, transferred to nitrocellulose membranes (Bio-Rad) and probed with primary antibodies, phosphoSTAT1(Tyr701) (Cell Signaling - #9171), STAT1 (Cell Signaling - #9172), phospho-Jak-1 (Santa Cruz – sc-101716) and Jak-1 (Santa Cruz – sc-7228). The anti-immunoglobulin G horseradish peroxidase conjugate (Bio-Rad) was used as the secondary antibody, and specific protein bands were visualized using the Luminata Forte Western HRP substrate (Millipore) following the manufacturer's suggested protocol.

### Immunoprecipitation

To decipher the p17 intracellular signalling THP-1 cells were serum starved over-night and then stimulated with p17 (1 µg/ml) for 15, 30 and 60 minutes. After the stimulation, cells were washed 3 times with ice-cold PBS and lysed with an insulin syringe in 500 µl E1A lysis buffer (250 mM NaCl, 50 mM Hepes pH 7.0, 0.1% NP40, 5 mM EDTA). Lysates were incubated 20 minutes in ice, clarified by centrifugation at 12000 rpm for 20 minutes at 4°C and quantified with Bradford reagent (PIERCE). 200 µg total proteins were pre-cleared on a rotating wheel for 1 h at 4°C using protein A Sepharose beads (Amersham Biosciences) and 2 µg of irrelevant antibody of the same species and isotype as RACK-1 (IgG2a (7D5) antibody (Santa Cruz - sc-69917)). Immunoprecipitation was performed overnight at 4°C with 1 µg RACK-1 antibody (Santa Cruz – sc-17754) or IgG as a negative control antibody in the presence of 40 µl of protein A Sepharose (Amersham Biosciences). The resultant immunoprecipitates were washed five times with 1 ml of lysis buffer and then used for western blotting using the antibodies syndecan-2 (Santa Cruz – sc-365624), Jak-1 and RACK-1.

### Chromatin Immunoprecipitation (ChIP)

THP1 cells serum starved over-night were stimulated for 1, 6 and 18 hours with p17 (1 µg/ml) were cross-linked with 1% formaldehyde at room temperature and the reaction was terminated by the addition of glycine to a final concentration of 0.125 M. Cells were washed in ice-cold PBS and lysed with SDS lysis buffer (1% SDS, 10 mM EDTA, and 50 mM Tris–HCl, pH 8). Cellular lysates were diluted with ChIP dilution buffer, sonicated, and immunoprecipitated with specific antibody anti-STAT-1 (Cell Signaling - #9172). Immunoprecipitates were collected with protein A beads (Amersham Bioscience) and washed sequentially first with a low-salt wash buffer and then with high-salt wash buffer using manufacturer's recommended procedures. DNA was eluted by addition of 1% SDS and 0.1 M NaHCO3, and the cross-linking reactions were reversed by heating the mixture to 65°C overnight. The DNA was recovered from immunoprecipitated material by proteinase K treatment at 65°C for 1 h followed by phenol/chloroform (1∶1) extraction, ethanol precipitation and dissolved into 50 µl of water. Five microliters of the extract was used for quantitative real-time PCR. Raw data analysis was performed as follows: ΔCt was calculated versus the input DNA concentration; ΔΔCt was versus unstimulated cells immunoprecipitated with the anti-IgG antibody (experimental condition set as 1); the relative expression was calculated as 2−(ΔΔCt).The sequences of primers used for the amplification of the MCP1 promoter [56] and the genomic region of FXR [30] containing a STAT-1 responsive element were: MCP1: cccatttgctcatttggtct and cttattgaaagcgggcagag; FXR genomic: catgaccaaggtagatcatgac and cccaagatacgtgcttgcat.

### Statistical analysis

All values are expressed as the mean ± SE of n observations per group. Comparisons of more than 2 groups were made with a one-way analysis of variance with post-hoc Tukey's tests. Differences were considered statistically significant if p was <0.05.

## Supporting Information

Method S1 **Detection of p17 antibody in blood samples.**
(DOC)

Figure S1 **Measurement of p17 antibodies in the serum of healthy donors (H1 and H2) and HIV infected patient before and after vaccination with a anti-p17 vaccine (pre-V and post-V).**
(PPT)
