# Supplementary material for: The HIV Matrix Protein p17 Subverts Nuclear Receptors Expression and Induces a STAT1-Dependent Proinflammatory Phenotype in Monocytes
Source: PLoS One. 2012 Apr 30;7(4):e35924. doi: 10.1371/journal.pone.0035924 (PMC3340403; doi:10.1371/journal.pone.0035924)
Supplement: Method S1 — Detection of p17 antibody in blood samples. (DOC) [file pone.0035924.s001.doc]

**Method S1.** Detection of p17 antibody in blood samples

A 96 wells plate was coated with p17 recombinant protein (400 ng/well) and incubated at 37 °C for 1 hour. The p17 coated plate was washed three times (3 minutes each) by flooding the wells with PBS 0.05% Tween 20. After washes wells were incubated with 200 μl of carbonate buffer 0.1 M pH 9.6 containing 5% BSA for 1 hour at 37 °C and washed three times with PBS 0.05% Tween 20. Serial dilutions of sera (1:10, 1:50, 1:100, 1:200, 1:400, 1:800, 1:1600, 1:3200, 1:6400 and 1:12800) were prepared in PBS and 200 μl of each dilution added into A1-A10, B1-B10, C1-C10 and D1-D10 wells and incubated at 37 °C for 1 hour. Positive and negative controls were plated into E1-E2, F1-F2, G1-G2, H1-H2 wells. After sera incubations, plates were washed three times with PBS 0.05% Tween 20 and incubated with 200 μl anti-human immunoglobulin conjugated with HRP (1:1000 in PBS 1% BSA) for 1 hour at 37°C. After three washes in PBS 0.05% Tween 20 wells were incubated with 200 μl substrate (5-ASA/H_2_O_2_). To prepare the substrate 5-aminosalycilic was freshly prepared by dissolving 80 mg of the acid in 100 ml of hot distilled water (80°C). This solution was cooled and stored at 4°C. Immediately before use, a portion of the solution was warmed to room temperature and the pH was brought to 6.0 with 1M NaOH. To 9 parts of 5-ASA, 1 part of 0.05% H2O2 (wt/vol) was added. The brown reaction product was evaluated visually and the results were measured by absorbance at 403 nm, which was determined in a GDV programmable MPT Reader (DV 990 BV4).
