# Supplementary material for: The HIV Matrix Protein p17 Subverts Nuclear Receptors Expression and Induces a STAT1-Dependent Proinflammatory Phenotype in Monocytes
Source: PLoS One. 2012 Apr 30;7(4):e35924. doi: 10.1371/journal.pone.0035924 (PMC3340403; doi:10.1371/journal.pone.0035924)
Supplement: Figure S1 — Measurement of p17 antibodies in the serum of healthy donors (H1 and H2) and HIV infected patient before and after vaccination with a anti-p17 vaccine (pre-V and post-V). (PPT) [file pone.0035924.s002.ppt]

## Slide 1
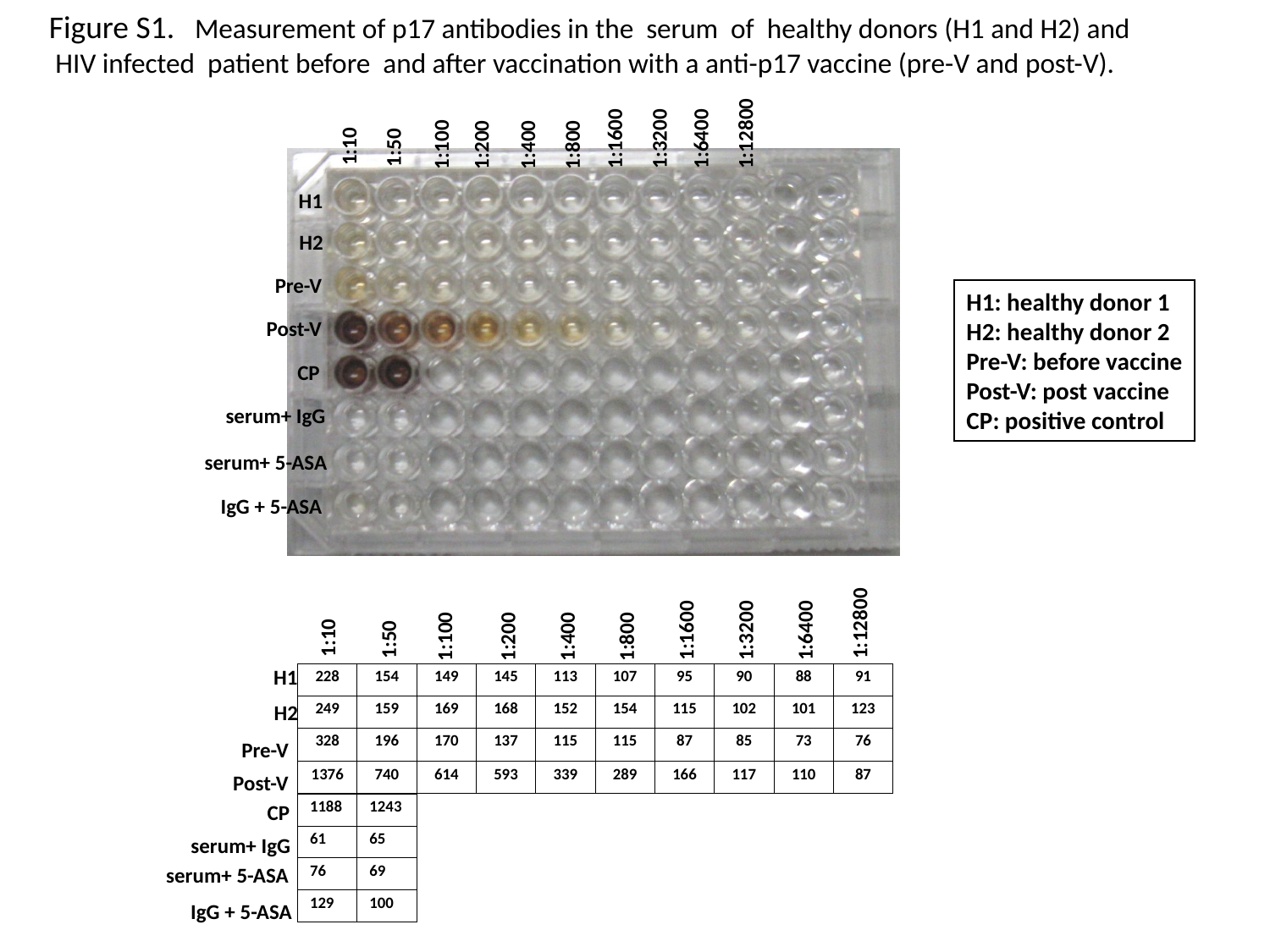

Figure S1. Measurement of p17 antibodies in the serum of healthy donors (H1 and H2) and
 HIV infected patient before and after vaccination with a anti-p17 vaccine (pre-V and post-V).
1:12800
1:1600
1:3200
1:6400
1:100
1:200
1:400
1:800
1:10
1:50
H1
H2
Pre-V
H1: healthy donor 1
H2: healthy donor 2
Pre-V: before vaccine
Post-V: post vaccine
CP: positive control
Post-V
CP
serum+ IgG
serum+ 5-ASA
IgG + 5-ASA
1:12800
1:1600
1:3200
1:6400
1:100
1:200
1:400
1:800
1:10
1:50
H1
| 228 | 154 | 149 | 145 | 113 | 107 | 95 | 90 | 88 | 91 |
| --- | --- | --- | --- | --- | --- | --- | --- | --- | --- |
| 249 | 159 | 169 | 168 | 152 | 154 | 115 | 102 | 101 | 123 |
| 328 | 196 | 170 | 137 | 115 | 115 | 87 | 85 | 73 | 76 |
| 1376 | 740 | 614 | 593 | 339 | 289 | 166 | 117 | 110 | 87 |
H2
Pre-V
Post-V
CP
| 1188 | 1243 |
| --- | --- |
| 61 | 65 |
| 76 | 69 |
| 129 | 100 |
serum+ IgG
serum+ 5-ASA
IgG + 5-ASA
